# Distinct biological effects of different nanoparticles commonly used in cosmetics and medicine coatings

**DOI:** 10.1186/2045-3701-1-19

**Published:** 2011-05-19

**Authors:** Julia X Yu, Thomas H Li

**Affiliations:** 1 Department of Cell Biology, Harvard Medical School, 240 Longwood Ave. Boston, MA. 02115. USA

## Abstract

**Background:**

Metal oxides in nanoparticle form such as zinc oxide and titanium dioxide now appear on the ingredient lists of household products as common and diverse as cosmetics, sunscreens, toothpaste, and medicine. Previous studies of zinc oxide and titanium dioxide in non-nanoparticle format using animals have found few adverse effects. This has led the FDA to classify zinc oxide as GRAS (generally recognized as safe) for use as a food additive. However, there is no regulation specific for the use of these chemicals in nanoparticle format. Recent studies, however, have begun to raise concerns over the pervasive use of these compounds in nanoparticle forms. Unfortunately, there is a lack of easily-adaptable screening methods that would allow for the detection of their biological effects.

**Results:**

We adapted two image-based assays, a fluorescence resonance energy transfer-based caspase activation assay and a green fluorescent protein coupled-LC3 assay, to test for the biological effects of different nanoparticles in a high-throughput format. We show that zinc oxide nanoparticles are cytotoxic. We also show that titanium dioxide nanoparticles are highly effective in inducing autophagy, a cellular disposal mechanism that is often activated when the cell is under stress.

**Conclusion:**

We suggest that these image-based assays provide a method of screening for the biological effects of similar compounds that is both efficient and sensitive as well as do not involve the use of animals.

## Background

As we move into an age of nearly complete dependence on artificial products and processes, we must be wary and mindful of the inherent risks of the goods we consume. There are many historical instances of faulty products being accepted too readily, spanning from the use of lead in piping and paint, mercury in pelt curing to the more recent introduction of thalidomide as a sedative and painkiller. Our experiment explores a potentially similar situation concerning some widely-used substances, namely a family of metal oxides in nanoparticle form. We are interested in compounds because of their pervasiveness in everyday life and their apparent low toxicity. Our experiment is designed to observe these nanoparticles via a number of image-based cellular assays which were not available or used when these substances were approved for general use.

Metal oxides such as zinc oxide (ZnO) and titanium dioxide (TiO_2_) in nanoparticle form have become extremely prevalent in day-to-day use. Zinc oxide and titanium dioxide now appear on the ingredients list of common household products as diverse as cosmetics, sunscreens, toothpaste, food coloring, paint and coatings for vitamin supplements. Titanium dioxide in particular is valued for its high refractive index and its bold white coloration, making it desirable as the most commonly used white pigment. It shares this quality with zinc oxide. Both zinc and titanium dioxide in nanoparticle form are common ingredients of sunscreen, as they are able to block out both UVA and UVB light. In normal particle form (>100 nm in size), however, they make the skin appear unsightly and white. Once a particle is reduced to nanoparticle size (0.2 nm - 100 nm), it begins to take on the properties of a finer particle. Sunscreens containing reflective metal nanoparticles cannot be seen when applied to the skin, but retain the reflective properties of larger particles.

Some prior studies had reported that micronized zinc oxide and titanium dioxide have no deleterious effects in studies of acute animal toxicity. A study conducted by Chen et al. concerning the incidence of lung cancer in workers exposed to titanium dioxide dust found no evidence of increased cancer risk associated with elevated exposure to titanium dioxide [1]. Another study conducted on mice concluded that feeding mice titanium dioxide-coated mica for 130 weeks caused no apparent toxic effects [2]. Such studies have led the FDA to classify zinc oxides as GRAS (generally recognized as safe) [3] and to approve titanium dioxide as an approved colorant for food, drugs, and medical devices. However, these studies, which were conducted on the level of whole beings, may not have been sensitive or extensive enough. Furthermore, these studies tested the properties of these chemicals in non-nanoparticle format; the chemicals in nanoparticle format might have distinct physical and biological activities.

Conclusions drawn from more recent research have begun to suggest that nanoparticles may be not as biologically inert as previously believed. A study conducted in 2009 concerning the effect of zinc oxide nanoparticles on neural stem cell apoptosis suggested that nano-sized zinc oxide would cause cell death when present in concentrations of 12 ppm or higher in a dose-dependent but not size-dependent manner [4]. This suggests that all nano-sized zinc is potentially dangerous, not only the super fine variety. Consistently, Hussain et al. observed that carbon black and titanium dioxide nanoparticles could induce apoptosis in bronchial epithelial cells [5]. Though both nanoparticle varieties in question caused apoptosis, the study concluded that the pathways taken were different: the carbon black nanoparticles induced apoptosis through a ROS-dependent mitochondrial pathway while the titanium dioxide nanoparticles induced a destabilization of the lysosomal membranes. This suggests that different nanoparticles may provoke different responses; this in turn suggests that responses triggered in different types of cells might also be different. A study examining the possibility of using quantum dot nanoparticles to label human mesenchymal stem cells was the first to introduce the possibility that nanoparticles could induce autophagy [6]. Together, these studies suggest that nanoparticles may exert certain biological effects in a context-dependent manner. The biological effects of the nanoparticles we feature in our experiment, however, have not been sufficiently examined.

The activation of caspases, a family of cysteine proteases, plays a critical role in mediating apoptosis [7]. As suggested by [4,5], nanoparticles may activate apoptosis. The assays used were, however, low-throughput western blots. They are therefore too inefficient to use to screen for apoptosis-inducing ingredients in common household products. An assay based on fluorescence resonance energy transfer (FRET) developed by Miura's lab represents a more efficient solution [8]. The assay was used to follow caspase activation in real time on the level of individual cells. We intend to use this assay to observe the biological effects of our nanoparticle compounds and judge its suitability as a method of high-throughput screening.

Autophagy is an important intracellular disposal mechanism that functions to remove and degrade expired or undesirable substances. Autophagy is often activated when cells are under stress [9]. Since experimental quantum dots have been suggested to induce autophagy in human mesenchymal stem cells [6], we would like to explore the possibility of other nanoparticles inducing autophagy as well and to examine the feasibility of using autophagy as a marker for screening the biological effects of nanoparticles.

A general goal of ours is to identify high-throughput assays that can be used routinely to screen nanoparticles for potentially harmful biological effects. Such screening would be instrumental in ensuring the safety of consumer products containing nanoparticles.

## Results

### FRET based high-throughput screen for caspase activation

To analyze the effects of nanoparticles on apoptosis, we decided to adapt two FRET-based molecular assays to detect caspase activation [8] using high-throughput microscopy. The indicator molecules for caspase activation use fluorescence resonance energy transfer (FRET) between an enhanced cyan fluorescent protein (ECFP, the donor) linked to an improved yellow fluorescent protein (Venus, the acceptor). The caspase-3 indicator (SCAT3) contains a caspase-3 cleavage site, i.e., the amino acid sequence DEVD (Asp-Glu-Val-Asp), which is cleaved by caspase-3 in stress-induced apoptosis. The caspase-9 indicator (SCAT9) contains a caspase-9 cleavage site, i.e., the amino acid sequence LEHD (Leu-Glu-His-Asp). Whether the linkage chain remains intact or not depends on caspase-3 or caspase-9 activity. If apoptosis is not induced when the compound in question is added to our seeded cells, the amino acid linkage chain remains intact. Venus will absorb the light emitted by ECFP and it will emit its own 530 nm light. If apoptosis is induced, caspase-3 and/or caspase-9 will be activated and their respective amino acid linkage chains will be cleaved. This severs the linkages between the fluorophores. The 475 nm light emitted by ECFP will not be absorbed by Venus and will instead be visible; consequently, Venus' 530 nm wavelength light will not be observed. The FRET signal is measured as a ratio of 530 nm light emitted to 475 nm light emitted (Venus:ECFP).

**Figure 1:**
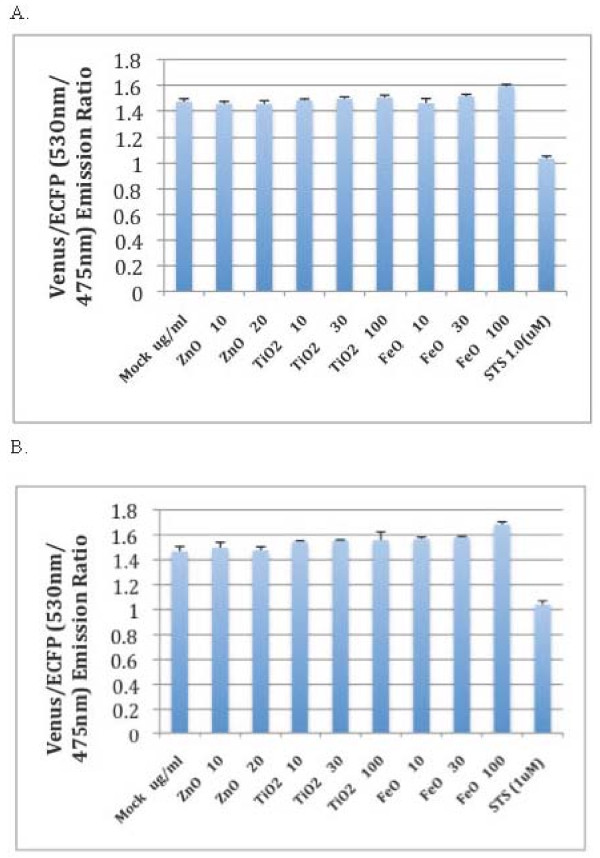
**Treatment with ZnO, TiO**_**2**_**, and FeO did not activate caspase-3 or caspase-9**. **A**, HT29-SCAT3 cells were treated with different nanoparticles as indicated (ZnO 10 = 10 μg/ml, ZnO 20 = 20 μg/ml, TiO_2 _10 = 10 μg/ml, TiO_2 _30 = 30 μg/ml, TiO_2 _100 = 100 μg/ml, FeO 10 = 10 μg/ml, FeO 30 = 30 μg/ml, FeO 100 = 100 μg/ml). Staurosporine (STS = 1 μM) was used as a positive control to induce caspase-3 activation. The images were analyzed using an automated ImageXpress Micro microscope at 20× magnification. The changes in the emission ratio after treatment and exposure to 435 nm light (530 nm ECFP/475 nm Venus) were measured as described by Takemoto et al. 2003 and quantitated using the MetaXpress software. This experiment was repeated 3 times. **B**, HT29-SCAT9 cells were treated with different nanoparticles as indicated (ZnO 10 = 10 μg/ml, ZnO 20 = 20 μg/ml, TiO_2 _10 = 10 μg/ml, TiO_2 _30 = 30 μg/ml, TiO_2 _100 = 100 μg/ml, FeO 10 = 10 μg/ml, FeO 30 = 30 μg/ml, FeO 100 = 100 μg/ml). Staurosporine (STS = 1 μM) was used as a positive control to induce caspase-9 activation. The images were analyzed using an automated ImageXpress Micro microscope at 20× magnification. The changes in the emission ratio after treatment and exposure to 435 nm light (530 nm ECFP/475 nm Venus) were measured as described by Takemoto et al. 2003 and quantitated using the MetaXpress software. This experiment was repeated 3 times.

As displayed in Figures 1, after our treatment of HT29-SCAT3 and HT29-SCAT9 cells with zinc oxide, titanium dioxide, and iron oxide nanoparticles for 18 hrs, no caspase activation was observed. No significant amount of caspase activation was observed with longer incubation either (data not shown). The positive control, staurosporine, induced caspase activation as indicated by the loss of FRET (Figure 1); this indicated that the assay was working. This result differs from relevant research as [4,5] had reported that zinc oxide and titanium dioxide nanoparticles would induce apoptosis.

**Figure 2:**
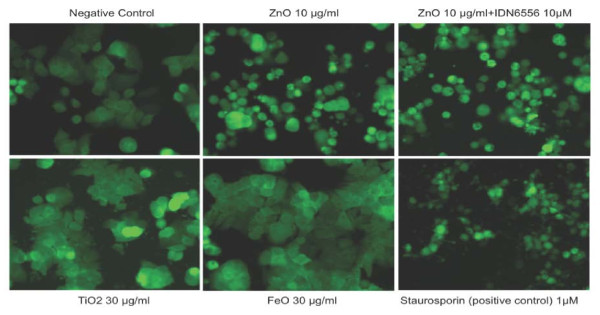
**Treatment with zinc oxide nanoparticles led to caspase-independent cell death**. HT29-SCAT3 cells were treated with 0.1% DMSO (vehicle; negative control), ZnO (10 μg/ml), TiO_2 _(30 μg/ml), FeO (30 μg/ml) and staurosporine (1 μM; positive control) for 18 hrs. The images were collected by an automated high-throughput microscope with a 20× objective (ImageXpress Micro). The addition of a caspase inhibitor IDN6556 (10 μM) did not inhibit cell death induced by ZnO. This experiment was repeated 3 times.

Next, we examined the morphologies of the treated cells. Although cells treated for 18 hours with up to 100 μg/ml of titanium dioxide and iron oxide nanoparticles appeared largely normal (data not shown), the cells treated with zinc oxide nanoparticles rounded up and died after 18 hours of incubation with 5-20 μg/ml zinc oxide (Figure 2). Furthermore, cell death induced by zinc oxide nanoparticles could not be inhibited by IDN6556, a potent and specific caspase inhibitor that has been shown to inhibit all caspases [10]. This is consistent with our FRET assay results, which indicate that zinc oxide nanoparticles cannot induce caspase activation. Our results suggest that zinc oxide nanoparticles induce cell death through a non-apoptosis pathway. Existing evidence suggests that the cells have multiple regulated pathways to mediate their death [11]. The alternative mechanisms of regulated cell death are currently a subject of intensive study. This part of our study demonstrates the feasibility of using FRET-based caspase activation assay in a high-throughput format to examine the pro-apoptotic activities of nanoparticles as well as other commonly used compounds.

### LC3-GFP-based high-throughput screen for autophagy activation

**Figure 3:**
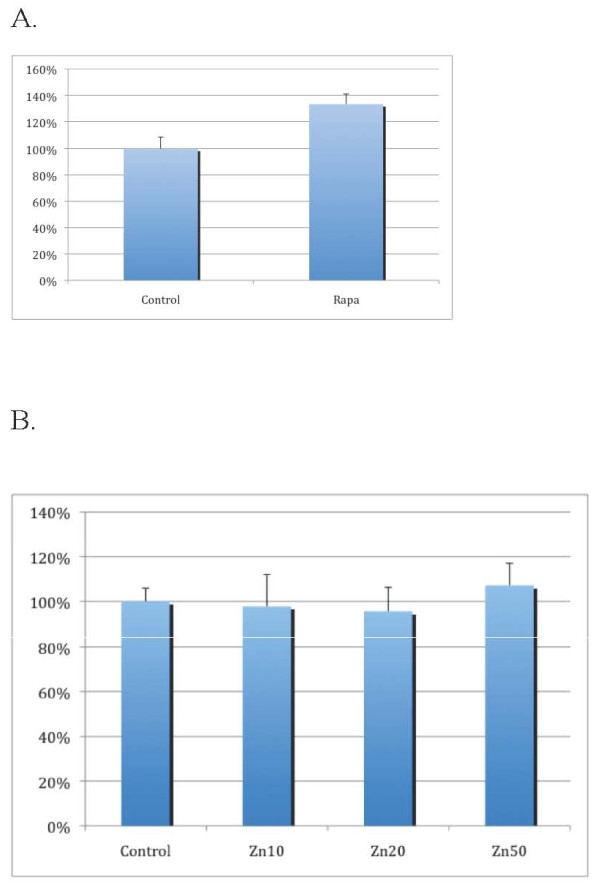
**Treatment with zinc oxide nanoparticles did not induce autophagy**. **A**, Treatment with rapamycin induced autophagy as a positive control. H4-LC3-GFP cells were treated with rapamycin (0.2 μM) for 18 hrs. The nuclei were stained with Hoechst dye. The images were analyzed with a high-throughput microscope CellWoRx with a 10× objective. The average area of LC3-GFP quata are shown. The treatment of rapamycin led to an increase in autophagy. P < 0.001. Student T test. **B**, H4-LC3-GFP cells were treated with zinc oxide nanoparticles (Zn10 = 10 μg/ml, Zn30 = 30 μg/ml, Zn100 = 100 μg/ml) as indicated for 18 hrs. The nuclei were stained with Hoechst dye and the LC3-GFP dots were quantified using CellWoRx microscope with a 10× objective. The average intensities of LC3-GFP dots are shown. This experiment was repeated 2 times.

Because autophagy is often activated in stressed cells [9], we considered the possibility that nanoparticles would induce autophagy. Induction of autophagy would not necessarily result in the death of the cell, but it would nevertheless indicate a stress response. To determine the effects of nanoparticles on autophagy induction, we used human neuroblastoma H4 cells stably expressing the LC3-GFP reporter [12]. LC3 is an important signaling molecule involved in mediating autophagy. When LC3 is activated, it is tagged with a small lipid, PE (phosphatidylethanolamine), that allows it to be translocated onto an autophagosomal membrane. In H4-LC3-GFP cells, LC3 has been tagged with a green fluorescent protein (GFP) to allow LC3 to be easily detected using fluorescent microscopy. Under normal conditions, LC3 is mostly present in the cytosol of a cell. When autophagy is activated, cytosolic LC3 (LC3 I) is conjugated into PE to form LC3 II which then translocates to the preautophagosomal membrane. We first tested using rapamycin as a positive control, as rapamycin is known to strongly induce autophagy [13]. Figure 3A shows that treatment of the cells with rapamycin, our positive control, strongly increased levels of observed autophagy. This indicates that our assay was working.

**Figure 4:**
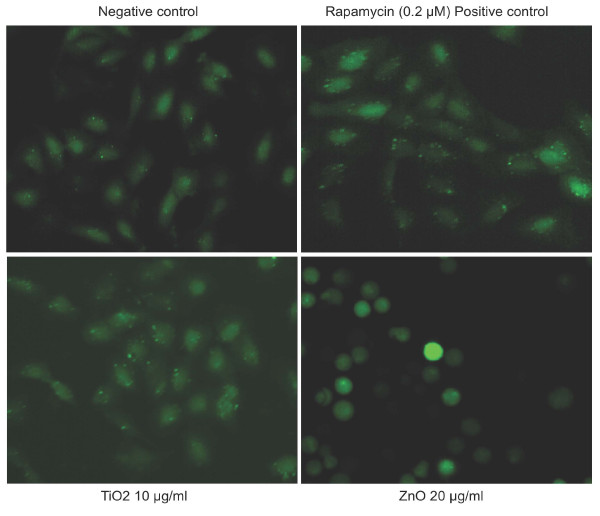
**Titanium dioxide nanoparticles but not zinc oxide nanoparticles induced autophagy**. H4-LC3-GFP cells were treated with zinc oxide (20 μg/ml) or titanium dioxide nanoparticles (10 μg/ml) for 18 hrs. 0.5% DMSO was used as a negative control. Rapamycin (0.2 μM) was used as a postive control. The images were recorded using a high-throughput microscope CellWoRx with a 10× objective. This experiment was repeated 2 times.

**Figure 5:**
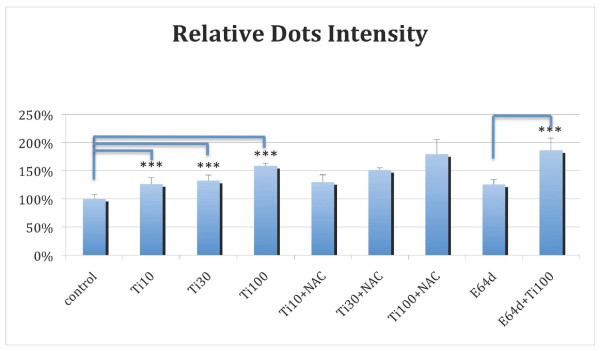
**Treatment with titanium dioxide nanoparticles led to increases in the autophagosome flux**. H4-LC3-GFP cells were treated with titanium dioxide nanoparticles (Ti10 = 10 μg/ml, Ti30 = 30 μg/ml, Ti100 = 100 μg/ml) both in the absence and presence of N-acetylcysteine (2.5 mM) or E64d (5 μg/ml) as indicated for 18 hrs. The nuclei were stained with Hoechst dye and the LC3-GFP dots were recorded using a high-throughput microscope CellWoRx with a 10× objective. The average intensities of LC3-GFP dots are shown. The images were quantified using VHSscan and VHSview image analysis software (Cellomics). The differences between control and Ti10, Ti30 or Ti100 and that of E64d and E64d + Ti1100, are highly significant with p < 0.0001 (***, Student T-test). This experiment was repeated 2 times.

On the other hand, although treatment with zinc oxide nanoparticles also induced death in H4-LC3-GFP cells as it did in HT29 cells, zinc oxide nanoparticles had no effect on autophagy (the number of green LC3-GFP dots did not increase) (Figure 3B &4). Thus, although zinc oxide nanoparticles induced cell death, it did not induce autophagy in H4 cells. Treatment with iron oxide also did not induce autophagy (data not shown). In contrast, we found that the treatment of the cells with titanium dioxide nanoparticles in concentrations that had no effect on cell morphology clearly led to significant increases in the levels of autophagy (Figures 4 and 5).

The accumulation of free radicals often plays a role in mediating autophagy [14]. Our next step was to investigate the involvement of free radicals in titanium dioxide induced autophagy. We soon found, however, that the addition of N-acetylcysteine (NAC), an antioxidant, did not inhibit the increases in autophagy induced by titanium dioxide nanoparticles (Figure 5). This suggests that the increases in autophagy induced by titanium dioxide nanoparticles may not be mediated by an increase in free radicals.

Because autophagosomes eventally fuse with lysosomes to degrade the contents of the autophagosome via hydrolytic enzymes, we had to consider the possibility that the apparent increases in the levels of autophagy may be due to a block in the lysosomes induced by titanium dioxide nanoparticles. The standard method to test this possibility is to use a blocker of lysosomes and to see if the combination of the lysosomal inhibitor with the compound of interest can lead to an additive increase in the level of autophagy [15]. We used E64d ([2S, 3S]-trans-Epoxysuccinyl-L-leucylamido-3-methylbutane ethyl ester), a cysteine protease inhibitor commonly used to inhibit lysosomal degradation [15]. We found that the combination of E64d and titanium dioxide led to a level of autophagy greater than that observed when E64d was applied alone, suggesting that the treatment of titanium dioxide led to increases in the flux of autophagy.

## Discussion

In our study, we adapted a number of image-based cell-based assays in high-throughput format to examine the possible biological effects of the nanoparticles that have become very common. Our study demonstrates the feasibility of providing side-by-side comparisons to examine the biological effects of compounds to which people are casually exposed. We demonstrate the advantage of image-based cellular assays that are sensitive and efficient as well as do not involve the use of animals. The high-throughput format of our assays makes them suitable as potential screening methods of choice for current and future consumer products of a similar nature. Our assays demonstrated the clear biological effects of nanoparticles at 0.001% to 0.01% final concentrations. These concentrations significantly lower than what the FDA has listed for these chemicals as safe. The FDA has approved the use of zinc oxide and titanium oxide "at concentrations of up to 25 percent alone and 2 to 25 percent in combination with any proposed Category I sunscreen active ingredient" [3] without specifications for the format of these chemicals. We have found certain sunscreens may contain up to 7% of zinc oxide or titanium dioxide nanoparticles.

Our results demonstrate that different nanoparticles may exert distinct biological effects. Contrary to earlier reports that titanium dioxide nanoparticles induce apoptosis [4,5], in our study, neither zinc oxide nor titanium dioxide nanoparticles induced apoptosis. Although zinc oxide did exhibit significant cytotoxicity, it did not induce the activation of caspases. Thus, we must consider the possibility that the cytotoxicity of zinc oxide nanoparticles may be mediated through apoptotic and non-apoptotic pathways. Titanium dioxide nanoparticles, on the other hand, caused significant levels of autophagy in concentrations that displayed no apparent cytotoxicity. We showed that autophagy induced by titanium dioxide nanoparticles is most likely through upstream activation, as coupling it with E64d, a blocker of lysosomal proteases, led to an increase in the level of observed autophagy. Because autophagy is a cellular disposal mechanism for removing unwanted material, the cells we used may have recognized titanium dioxide nanoparticles as foreign. Autophagy may have been induced as an effort to get rid of these nanoparticles.

Our results are highly relevant for the safety of common consumer products. Because nanoparticles appear most commonly in sunscreens, sunscreens have been the subject of most previous research on the effect of the nanoparticles in our experiment. Some studies conclude that nanoparticles are unlikely to pass through the upper layer of the skin, the stratum corneum, after being coated in manufacturing [16]. Other than the issue of the sensitivity of the assays used, these studies focus largely on healthy human skin. The fact remains that damaged skin, whether previously UV-damaged, dry, or otherwise compromised skin, is significantly more sensitive to lotions and creams applied topically. Such previous studies have dismissed the possibility of a damaged upper layer of skin on a sunscreen user. A simple sunburn is known to damage or peel the stratum corneum, and a sunburned individual is likely to attempt to prevent this condition from worsening by applying additional sunscreen. A damaged stratum corneum would likely allow for exposure of viable living cells and subsequent penetration by nanoparticles in sunscreen. Further testing in this field is needed.

Skin lotion, often applied on damaged, dry, or otherwise imperfect skin, is another source of metal oxide nanoparticles. A major cosmetic company with an interest in nanotechnology has a product line that includes sunscreens, hair conditioners, and skin lotions. Along with lotions including micro- or nanosized particles of titanium dioxide, it has reportedly developed nanocapsules capable of "guiding active ingredients into the lower levels of the skin." While this already opens viable skin cells to the possibility of exposure to nanoparticles, it is important to note that some of this company's creams list titanium dioxide as an active ingredient (up to 10% content). Moisturizing creams designed for daytime use also often incorporate a sun protection factor (SPF), which entails the incorporation of nanoparticles. The earlier studies emphasizing these nanoparticles as harmless due to their inability to penetrate the outer layer of healthy skin may have reached premature conclusions. The logical course of action is to repeat the studies outlined in this report on both damaged and viable skin cells.

Vitamin supplements manufactured by many companies are labeled as containing titanium dioxide and zinc oxide in nanoparticle form as additives or coating. The risks of this form of contact are especially relevant to this report, as HT29 (human colon adenocarcinoma cells) cells were one of the cell types used. Once ingested, the supplement is broken down and any additives are carried into the body. Once the digested material enters the intestinal tract, the sensitive lining of the colon is exposed to any nanoparticle dust present in the stool.

Toothpaste is another source of nanoparticles that can be potentially exposed to colon cells. While toothpaste is not designed to be swallowed, studies have shown that young children (<6 years of age) tend not to be in full control of the swallowing reflex. Along with harmful amounts of fluoride, children in a delicate stage of physical and mental development may be subject to irreversible cell damage due to nanoparticle exposure.

The FDA currently lists 3 chemicals examined in our experiment as generally safe for use in food products without specifications for the formats of these chemicals. Since these regulations were established before the development of nanotechnology, it is time to carefully examine these issues and update the regulation. We have shown that zinc oxide and titanium dioxide nanoparticles in concentrations as low as 10 μg/ml (0.001% by weight) can clearly induce cytotoxicity or a stress response. The results of our experiment suggest that nanoparticles may exert biological activities that are not shared in non-nanoparticle format.

## Conclusions

Our study observes the distinct biological effects of common metal oxide nanoparticles. We have shown that zinc oxide nanoparticles exhibit significant cytotoxicity which may be mediated through non-apoptotic cell death mechanisms. We have also shown that titanium dioxide nanoparticles can induce autophagy through upstream signal activation. Because both zinc oxide and titanium dioxide nanoparticles exhibit distinct biological effects, further studies are needed to address the exact mechanisms used to explain how different nanoparticles interact with or disrupt cellular processes.

We have also demonstrated the feasibility of using high-throughput image-based cellular assays to test the biological effects of different nanoparticles as well as other compounds used in common consumer products. Because these assays are highly sensitive, they are able to detect biological effects at levels much lower than those detectable by animal studies. Furthermore, our studies provide a method alternative to animal testing. Another clear advantage of such high-throughput assays compared to the assays used in earlier studies [1,2,4,5], is that they can be standardized and automated to provide side-by-side comparisons of different substances of interest. Once the instrument (high-throughput microscopes) and the associated software are installed, the experiments and image analysis do not require additional reagents other than cell culture media. The operation is also relatively simple.

Taken together, our studies demonstrate that nanoparticles are not biologically inert as widely believed. We urge further careful studies on these nanoparticles for their safety as common consumer products. Our lives may be adversely affected by using the very same products that were designed to protect us from our environment.

## Methods

### 1. FRET assay

We adapted a FRET-based assay originally developed by Miura's lab in Japan to screen for apoptosis [8]. The cells used were HT29 human colon adenocarcinoma cells stably expressing FRET-based reporters for caspase-3 and caspase-9: SCAT3 and SCAT9, respectively. The FRET signal is measured as a ratio of 530 nm light emitted to 475 nm light emitted (Venus:ECFP). The images taken of our assay were collected by an automated high-throughput microscope (ImageXpress Micro made by Molecular Devices) and analyzed with MetaXpress software.

### 2. High-throughput LC3-GFP imaging analysis

H4-LC3-GFP cells were used to determine the levels of autophagy. The number, size and intensity of the green dots in H4-GFP cells indicate the levels of autophagy in the cell. Different autophagy inducers may affect the size, number or intensity of the LC3-GFP green dots in different ways [17]. Our cells were imaged on an automated CellWoRx microscope (made by Applied Precision) at 10× magnification and 350 nm (Hoechst) and 488 nm (LC3-GFP) wavelengths. All images were quantified using VHSscan and VHSview image analysis software (Cellomics). The software scored total cell number, total and intensity of LC3-GFP, as well as the number, area, and intensity of LC3-GFP positive autophagosomes.

### 3. Cell culture

Our sample cells were HT29 human colon adenocarcinoma cells and H4 human gliobastoma cells cultured in DMEM plus 10% fetal bovine serum. For H4 cells, 1 mM sodium pyruvate was also added to the medium. The cells used for imaging were plated in 96-well plates with 6,000 cells in each well. Each data point represents an average of at least 3 individual wells.

### 4. Chemical sources

Zinc oxide nanoparticles (catalog No. 721077, average size <35 nm), titanium dioxide nanoparticles (catalog No. 637254, average size <25 nm), iron oxide nanoparticles (catalog No. 720704, average size <30 nm) and E64d (E8640) were purchased from Sigma-Aldrich. IDN6556 was obtained from TetraLogic Pharmaceuticals, Inc. Stock suspensions of the NPs were made at a concentration of 2 mg.ml^-1 ^in culture media and diluted into appropriate concentrations with pipetting. Care was taken to make sure no visible aggregates under microscope.

## Competing interests

The authors declare that they have no competing interests.

## Authors' contributions

JY and TL conducted all of the experiments described in this manuscript. JY wrote the manuscript and edited by TL. All authors have read and approved the final manuscript.
